# Unmasking Distal Renal Tubular Acidosis in a Young Female: A Case of Metabolic Acidosis With Hypokalemic Paraparesis

**DOI:** 10.7759/cureus.85193

**Published:** 2025-06-01

**Authors:** GKM Rashik Uzzaman, Muhammad Mohsin Isar, Sultana Jannatun Nahar, Naeem Rana, Taslima Moonmoon

**Affiliations:** 1 Acute Medicine, United Lincolnshire Hospitals NHS Trust, Lincoln, GBR; 2 Acute Medicine, United Lincolnshire NHS Hospital Trust, Boston, GBR; 3 General Medicine, United Lincolnshire NHS Hospital Trust, Boston, GBR

**Keywords:** distal renal tubular acidosis, electrolyte disturbances, flaccid weakness, non-anion gap metabolic acidosis, severe hypokalemia induced paralysis

## Abstract

This case report aims to highlight the diagnostic challenge and clinical significance of type 1 distal renal tubular acidosis (dRTA) in young patients presenting with hypokalemic paralysis. A 19-year-old female presented with vomiting, progressive lower limb weakness, and severe hypokalemia. Investigations revealed normal anion gap metabolic acidosis, elevated urinary pH, and renal potassium loss. After ruling out other causes, a diagnosis of dRTA was made. She responded well to potassium and bicarbonate therapy. Early recognition and treatment are essential to prevent complications and ensure full recovery.

## Introduction

Renal tubular acidosis (RTA) is a condition characterized by a defect in renal tubular function, leading to the inability of the kidneys to acidify the urine. This results in systemic metabolic acidosis despite a normal glomerular filtration rate. RTA can be classified into different types, with type 1 (Distal), type 2 (Proximal), type 3 (Mixed), and type 4 (Hyperkalemic) [1,2]. This case report highlights the clinical features, diagnosis, and management of a patient with type 1 RTA, a rare but significant renal disorder.

Type 1 distal RTA (dRTA) results from the failure of the distal tubules to secrete hydrogen ions into the urine [3,4]. This leads to persistent metabolic acidosis, hypokalemia, and a failure to acidify urine to a pH below 5.5 despite systemic acidemia. dRTA is often associated with nephrocalcinosis, nephrolithiasis, bone demineralization, and growth retardation in children [5,6].

## Case presentation

A 19-year-old female presented to the hospital with a two-day history of generalized weakness, abdominal pain radiating to the lower back, and episodes of vomiting. Over the preceding week, she had experienced progressive difficulty in mobilizing, with marked weakness predominantly affecting the lower limbs. Her medical history was notable for hypothyroidism, managed with levothyroxine 100 mcg daily, and migraines treated with acetaminophen (paracetamol). She also reported a recent spontaneous miscarriage approximately one month prior.

On initial examination, she appeared mildly dehydrated with generalized abdominal tenderness. A neurological assessment revealed flaccid paraparesis with muscle power graded 2/5 in both lower limbs, reduced tone, absent deep tendon reflexes, and preserved sensory and sphincter function. Although the patient complained of generalized weakness, there was no evidence of upper extremity involvement; the weakness was confined to the lower limbs. These findings raised concern for a possible metabolic or neurological cause of acute motor weakness.

Her clinical observations at presentation included a pulse rate of 117 bpm, respiratory rate of 18/min, blood pressure of 142/71 mmHg, temperature of 36.8 °C, and oxygen saturation of 97% on room air.

Initial blood investigations (Table 1) were notable for a critically low serum potassium level of 1.85 mmol/L, low bicarbonate at 15.8 mmol/L, metabolic acidosis with a blood pH of 7.298, PCO₂ of 5.4 kPa, and base excess of -8.9 mmol/L. Serum chloride was elevated at 119 mmol/L, and the anion gap was 11, indicating a normal anion gap and hyperchloremic metabolic acidosis. This biochemical pattern suggested a non-anion gap acidosis, likely of renal or gastrointestinal origin. However, the presence of vomiting would typically cause metabolic alkalosis rather than acidosis, leading clinicians to suspect a renal tubular disorder. Given the severity of hypokalemia and its known association with neuromuscular symptoms, a renal cause for both the acidosis and potassium loss was investigated. Urinary studies (Table 1) further revealed a high urinary potassium concentration of 30 mmol/L, a positive urinary anion gap, and a persistently elevated urine pH of 6.0 despite systemic acidosis. Urinary glucose and amino acids were absent. These findings indicated inappropriate urinary acidification and ongoing renal potassium wasting, which are hallmark features of distal (type 1) renal tubular acidosis (dRTA).

**Table 1:** Initial blood and urine results (at presentation) showing type 1 (distal) renal tubular acidosis, persistent metabolic acidosis, hypokalemia, positive urinary anion gap, and failure to acidify urine despite acidosis PCO2: partial pressure of carbon dioxide, PH: potential of hydrogen, GFR: glomerular filtration rate, mmol/L: millimoles per liter, kPa: kilopascal, mL/min: milliliters per minute, pmol/L: picomoles per liter

Test / Parameter	Result	Units	Reference Range	Interpretation/ Notes
Potassium	1.85	mmol/L	3.5–5.0 mmol/L	Severe Hypokalemia
Bicarbonate	15.8	mmol/L	22–28 mmol/L	Low
Blood pH	7.298		7.35–7.45	Metabolic acidosis
PCO2	5.4	kPa	4.7–6.0 kPa	Normal
Base Excess	-8.9	mmol/L	-2 to +2 mmol/L	Low
Serum Chloride	119	mmol/L	98–106 mmol/L	High, hyperchloremic metabolic acidosis
Anion Gap	11		8–16	Normal
Urinary Potassium	30	mmol/L	25–125 mmol/L	Renal potassium loss
Urine pH	6.0		4.5–8.0	High, impaired acidification
Urinary Glucose	Absent		Absent	No glycosuria
Amino Acids	Absent		Absent	No aminoaciduria
Urinary Anion Gap	Positive		Negative to variable	
Serum Aldosterone	450	pmol/L	100-450 pmol/L	Normal
eGFR	>90	mL/min	>90 mL/min/1.73m²	Normal renal function

To further narrow the differential diagnosis, proximal RTA was excluded by the absence of glycosuria, aminoaciduria, and hypophosphatemia. Type 4 RTA was ruled out due to the presence of hypokalemia rather than hyperkalemia and normal aldosterone levels (Table 1). Chronic kidney disease was considered unlikely given the normal estimated glomerular filtration rate (eGFR >90 mL/min) and unremarkable imaging of the kidneys, with no signs of nephrocalcinosis or nephrolithiasis on ultrasound.

**Figure 1:**
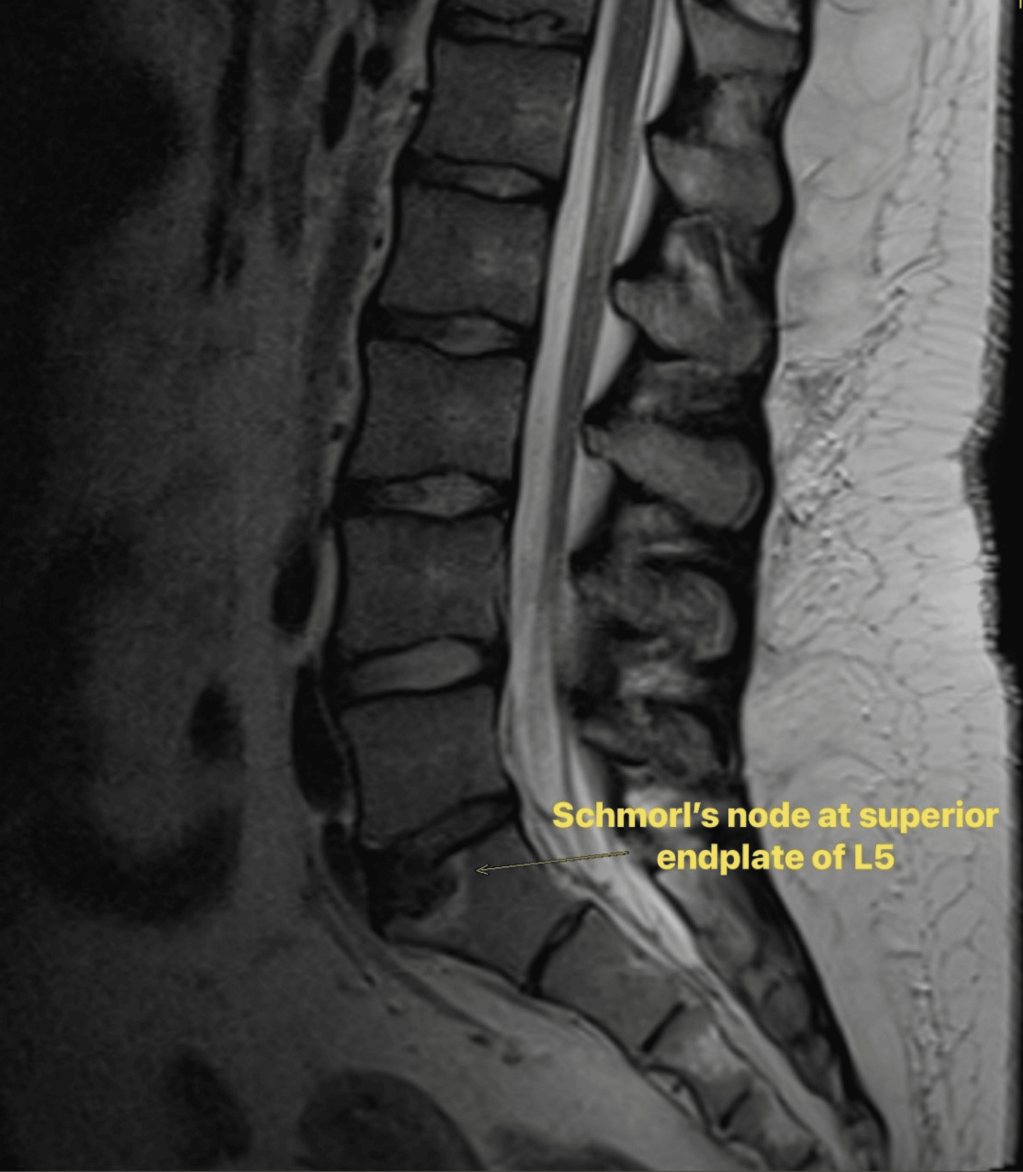
MRI spine showing Schmorl’s node at the superior endplate of L5

Due to her complaints of back pain and paresthesia in the legs, an MRI of the spine (Figure 1) was performed, which showed a transitional vertebra at the lumbosacral junction and Schmorl’s nodes but no evidence of spinal cord or cauda equina compression. This supported the conclusion that the neurological deficits were secondary to electrolyte disturbances rather than structural pathology.

**Figure 2:**
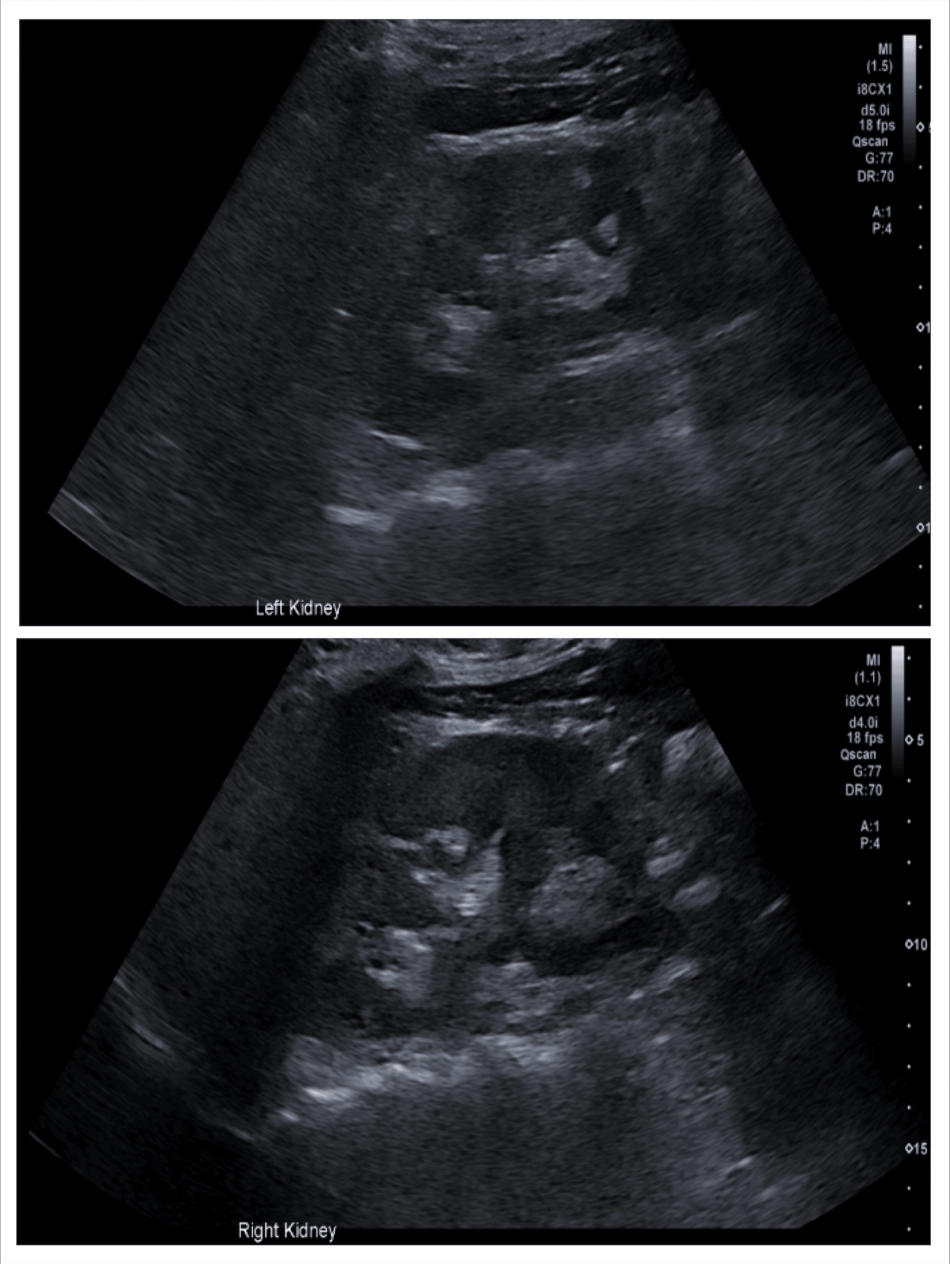
Ultrasound of kidneys showing the normal appearance of both kidneys

**Figure 3:**
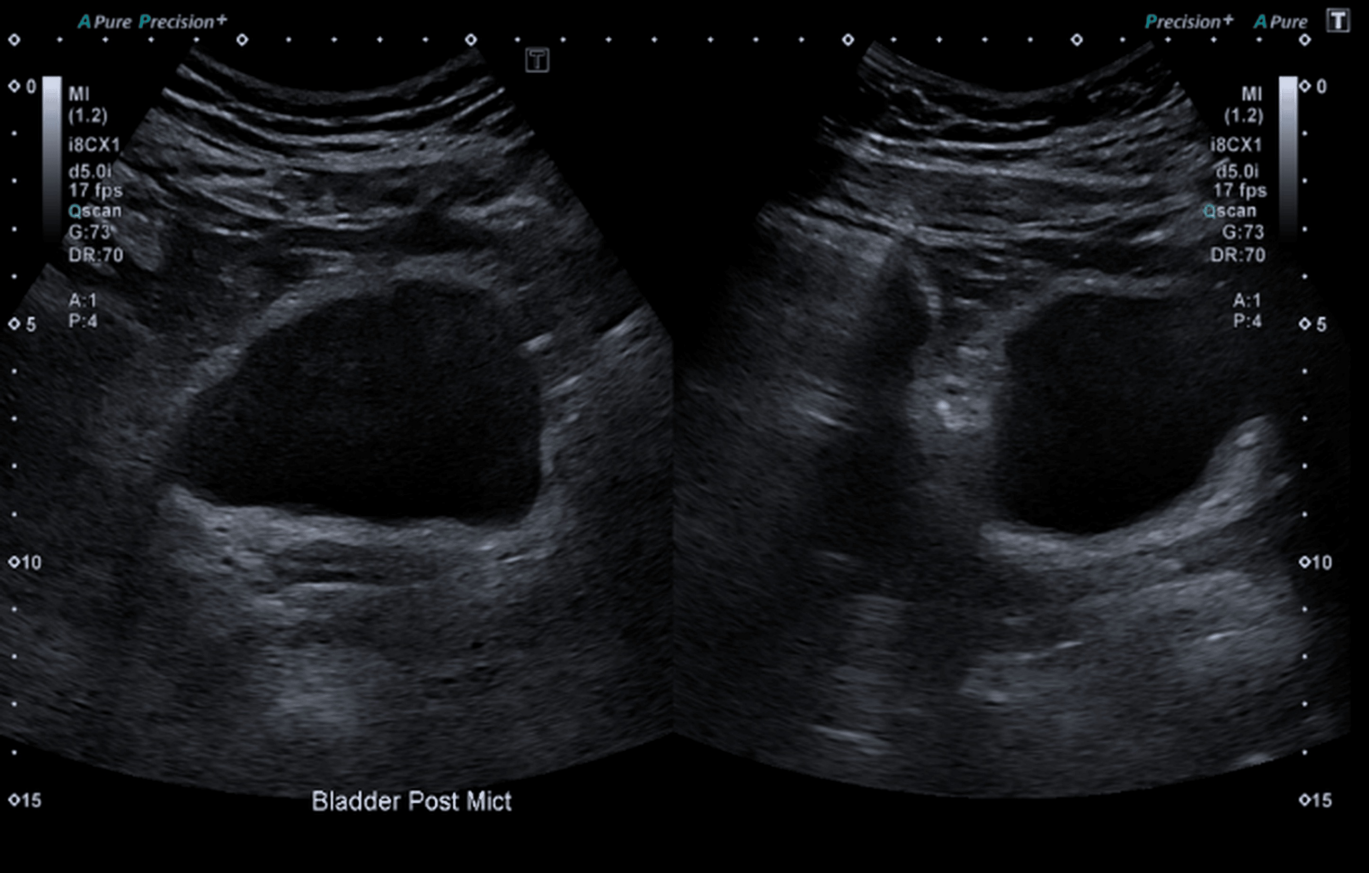
Ultrasound of the urinary bladder showing a normal appearance, although the bladder has a large post-void residual volume of 138 cc

Ultrasound of the kidneys (Figure 2) revealed normal appearances of both kidneys, and ultrasound of the bladder (Figure 3) showed normal appearance, although the bladder had a large post-void residual volume of 138 cc.

Taken together, the clinical presentation of muscle weakness and vomiting, biochemical evidence of normal anion gap metabolic acidosis with hyperchloremia, renal potassium wasting, high urinary pH, and exclusion of other differential diagnoses confirmed the diagnosis of type 1 (distal) RTA.

The patient was admitted under the nephrology team for targeted management. She was initially treated with intravenous potassium replacement due to the severity of her hypokalemia and subsequently transitioned to oral potassium supplements (potassium citrate). Oral sodium bicarbonate (1 g four times daily) was initiated to correct the underlying metabolic acidosis. Levothyroxine was continued as part of her ongoing hypothyroidism management. With treatment, her potassium and bicarbonate levels gradually stabilized, and her muscle strength improved significantly. Muscle strength in the lower limbs improved from an initial grade of 2/5 to 4/5 over the course of her inpatient stay. At the time of discharge, there were no residual neurological deficits, and upper limb strength remained normal throughout. Given the complete resolution of symptoms, no further neurological investigations were deemed necessary.

She remains under regular nephrology follow-up, and genetic testing was recommended to investigate a possible hereditary etiology, as her grandmother had reportedly experienced similar symptoms. The patient continues to be under regular follow-up in the nephrology clinic. During the follow-up investigations (Table 2), she maintained stable bicarbonate and potassium levels with ongoing oral supplementation.

**Table 2:** Results after treatment/during follow-up showing improved potassium and bicarbonate levels, stable with sodium and potassium replacements

Test / Parameter	Result	Units	Reference Range
Potassium	3.77	mmol/L	3.5–5.0 mmol/L
Bicarbonate	18	mmol/L	22–28 mmol/L
Blood pH	7.32		7.35–7.45
Serum Bicarbonate	15.8	mmol/L	22–28 mmol/L
Anion Gap	11		8–16
Serum Chloride	119	mmol/L	98–106 mmol/L

## Discussion

**Figure 4:**
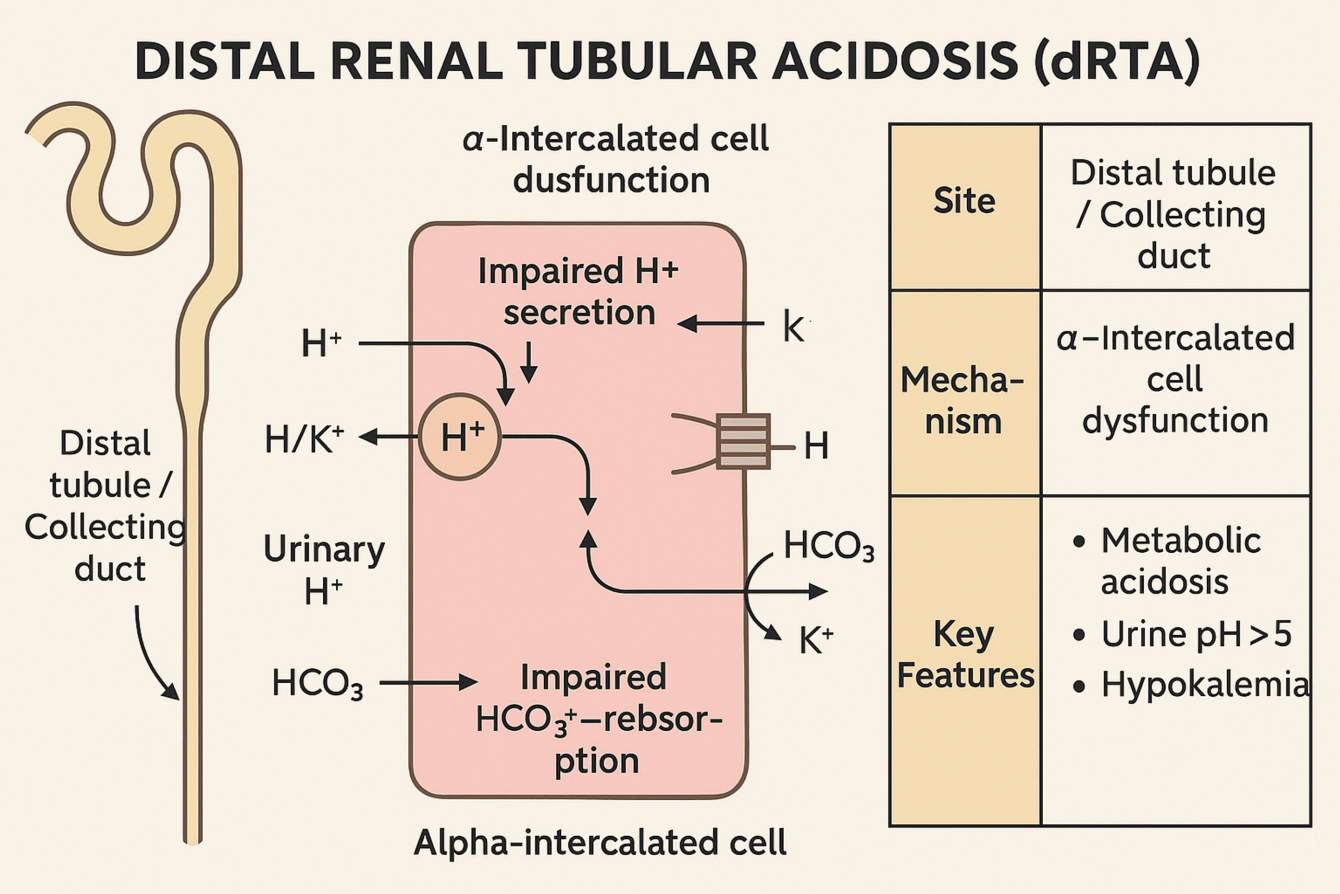
Pathophysiology of distal renal tubular acidosis Diagram illustrating the pathophysiology of distal renal tubular acidosis (dRTA), including alpha-intercalated cell dysfunction and impaired hydrogen ion secretion. Original figure created by the authors.

Type 1 distal renal tubular acidosis is a rare and clinically significant condition characterized by the inability of the distal renal tubules to secrete hydrogen ions. This defect leads to systemic metabolic acidosis with hypokalemia and a failure to acidify urine to a pH below 5.5 even during systemic acidosis. Unlike type 2 (proximal) RTA, distal RTA is primarily due to a defect in the alpha-intercalated cells of the collecting duct, which fail to secrete hydrogen ions properly, leading to bicarbonate loss and acid retention (Figure 4).

Pathophysiologically, the hallmark of dRTA is an inability to generate a sufficient hydrogen ion gradient, resulting in urinary pH remaining above 5.5 despite systemic acidosis [2,5,7]. Additionally, potassium wasting occurs due to enhanced potassium secretion in response to persistent acidemia. Chronic dRTA can lead to nephrocalcinosis, nephrolithiasis, osteomalacia, growth retardation, and chronic kidney disease if untreated [6].

This patient's clinical presentation of generalized weakness, abdominal pain, muscle weakness, and flaccid paraparesis is consistent with hypokalemia, a key manifestation of dRTA. Laboratory investigations confirmed metabolic acidosis with a normal anion gap, hyperchloremia, and hypokalemia. The persistently high urinary pH and positive urinary anion gap indicated renal tubular loss of potassium and an inability to excrete hydrogen ions effectively.

The differential diagnosis of RTA includes proximal (type 2) and hyperkalemic (type 4) RTA. Type 2 RTA was ruled out due to the absence of glycosuria, hypophosphatemia, and aminoaciduria (Table 3). Additionally, the normal serum aldosterone level excluded type 4 RTA, which is typically associated with hyperkalemia and aldosterone deficiency or resistance.

**Table 3:** Comparison of RTA type 1 (distal) and RTA type 2 (proximal) RTA: renal tubular acidosis

Feature	RTA Type 1 (Distal)	RTA Type 2 (Proximal)
Defect	Reduced H⁺ excretion in distal tubule	Impaired HCO₃⁻ reabsorption in the proximal tubule
Minimum urine pH	> 5.5	< 5.5
Plasma HCO₃⁻	< 15 mmol/L	Usually > 15 mmol/L
Serum potassium	Low (hypokalemia, often severe)	Low (hypokalemia, milder)
Renal stones	Yes	No
Urinary anion gap	Positive	Variable
Urinary potassium	High (renal wasting)	Variable
Urinary glucose/amino acids	Absent	May be present (Fanconi syndrome)
Urine acidification after acid load	No (urine pH remains >5.5)	Yes (urine pH falls <5.5)
Common causes	Autoimmune, hereditary, and drugs	Hereditary, drugs, and Fanconi syndrome
Bone involvement	Osteomalacia, rickets, and nephrocalcinosis	Osteomalacia (rare nephrocalcinosis)
Typical age group	Children and adults	Often children
Treatment	Alkali and potassium supplementation	Alkali (high dose), potassium

Treatment of dRTA primarily involves correction of metabolic acidosis and potassium supplementation, e.g., potassium citrate. Alkali therapy, using sodium bicarbonate or potassium citrate, is essential to neutralize acidosis and restore normal bicarbonate levels [5]. Potassium supplementation is also crucial to prevent hypokalemia, which can be life-threatening if not adequately managed.

The patient responded well to intravenous potassium citrate replacement during the acute phase and was subsequently maintained on oral potassium chloride syrup and bicarbonate therapy. Genetic testing is recommended in such cases, especially with a positive family history [5], as noted in this patient, to identify underlying hereditary causes.

## Conclusions

This case highlights the importance of considering distal renal tubular acidosis (dRTA) in young patients presenting with hypokalemia and metabolic acidosis, particularly when neuromuscular symptoms, such as flaccid paralysis, are evident. The biochemical profile of non-anion gap hyperchloremic acidosis and potassium loss was critical to diagnosis. Notably, hypokalemia in dRTA can manifest with neurological symptoms, including acute lower limb weakness, which may mimic spinal or neuromuscular pathology. Long-term management of dRTA requires ongoing monitoring of serum bicarbonate, potassium levels, renal function, and growth parameters. The prognosis is generally favorable with early diagnosis and appropriate treatment, but complications such as nephrocalcinosis, renal stones, and bone demineralization may develop if untreated.
